# Complete Genome Sequence of Pseudomonas putida BS3701, a Promising Polycyclic Aromatic Hydrocarbon-Degrading Strain for Bioremediation Technologies

**DOI:** 10.1128/MRA.00892-20

**Published:** 2020-10-01

**Authors:** Andrey Filonov, Yanina Delegan, Irina Puntus, Leonid Valentovich, Artur Akhremchuk, Olesia Evdokimova, Tatyana Funtikova, Marina Zakharova, Lenar Akhmetov, Anna Vetrova, Marina Titok

**Affiliations:** a Institute of Biochemistry and Physiology of Microorganisms, Pushchino, Moscow Region, Russian Federation; b Federal Research Center Pushchino Scientific Center for Biological Research of the Russian Academy of Sciences, Pushchino, Moscow Region, Russian Federation; c Tula State University, Tula, Russian Federation; d Institute of Microbiology of the National Academy of Sciences of Belarus, Minsk, Belarus; e Belarus State University, Minsk, Belarus; University of Arizona

## Abstract

The strain Pseudomonas putida BS3701 was isolated from soil contaminated with coke by-product waste (Moscow Region, Russian Federation). It is capable of degrading crude oil and polycyclic aromatic hydrocarbons (PAHs). The P. putida BS3701 genome consists of a 6,337,358-bp circular chromosome and two circular plasmids (pBS1141 with 107,388 bp and pBS1142 with 54,501 bp).

## ANNOUNCEMENT

Pseudomonads are known for numerous strains able to degrade various organic compounds, including both aliphatic and aromatic hydrocarbons (1). The biodegradation of polycyclic aromatic hydrocarbons (PAHs) is often controlled by plasmids, most of which are found in representatives of the genus *Pseudomonas* (2). The strain Pseudomonas putida BS3701 (plasmids pBS1141 and pBS1142) was isolated from soil contaminated with coke by-product waste (sewage from the Moscow Coke and Gas Plant, Vidnoye, Moscow Region, Russian Federation) (3). It is capable of degrading crude oil and PAHs (strain certificate VKM B-2380D). For long-term storage, the strain was kept in glycerol (40%) stocks at −70°C. For short-term maintenance, the strain was cultured on LB agar plates at 24°C.

Genomic DNA was isolated from a fresh culture biomass (a single colony) of Pseudomonas putida BS3701 grown on LB agar using a DNeasy blood and tissue kit (catalog number 69506; Qiagen). Sequencing was performed using a MinION sequencer (Oxford Nanopore Technologies [ONT]) at the Center of Analytical and Genetic Engineering Research (Minsk, Belarus). The library was prepared with a ligation sequencing kit (catalog number SQK-LSK109). Guppy v. 3.6.0 software was used for base calling.

Additionally, the same DNA sample was sequenced with an Illumina MiSeq platform using a MiSeq reagent kit v. 2 (2 × 251 bp). A paired-end library for sequencing was prepared with the MuSeek library preparation kit (catalog number K1361; Thermo Fisher).

The Nanopore reads were binned and filtered by quality (Q score, >10) using Barapost v. 2020-06-26 (4), which yielded a total of 159.24 Mbp distributed in 17,240 reads with *N*_50_/*N*_90_ values of 15,504/4,540 bp. Prepared reads were assembled into 3 contigs using Flye v. 2.7.1-b1590 (5). The contigs were checked for circularization using the Tablet software (6). The Illumina reads were assembled using SPAdes v. 3.14.1 (7). The Illumina reads were used to correct Nanopore errors using Bowtie 2 v. 2.3.5.1 (8) and Pilon v. 1.23 (9) software. Default parameters were used for all software.

The P. putida BS3701 genome consists of a 6,337,358-bp circular chromosome (GC content, 61.6%) and two circular plasmids. The first one, namely, pBS1141, has 107,388 bp and a GC content of 57.8%, and the second one, pBS1142, has 54,501 bp and a GC content of 57.4%. Chromosome and plasmid circularization was specified by ends overlapping.

The strain BS3701 genome was annotated with the NCBI Prokaryotic Genome Annotation Pipeline (PGAP) v. 4.6 (10). The chromosome contained 5,756 coding sequences, 7 rRNA clusters (5S, 16S, 23S), and 79 tRNAs. Previously (11), Pseudomonas putida BS3701 was shown to be capable of growth using naphthalene as the sole carbon and energy source. The genes of naphthalene degradation (naphthalene 1,2-dioxygenase large and small subunits, 2-hydroxymuconic semialdehyde hydrolase, catechol 2,3-dioxygenase, and salicylate 1-monooxygenase) were found on plasmid pBS1141. The mating experiment (12) indicated that pBS1142 is a helper plasmid, which could assist plasmid pBS1141 to transfer during conjugation.

The antiSMASH search for secondary metabolite clusters found 9 clusters on the main chromosome, including clusters of lipopolysaccharides and fengycin production (13). The genome sequence data of Pseudomonas putida BS3701 will enhance our understanding of the metabolic capabilities of *Pseudomonas* strains.

### Data availability.

This genome project has been deposited at GenBank under the accession numbers CP059052.1 for the chromosome, CP059053.1 for plasmid pBS1141, and CP059054.1 for plasmid pBS1142. Other data can be found under BioSample number SAMN15517094 and BioProject number PRJNA645807. The SRA accession numbers are SRX8724918 (Illumina) and SRX8724919 (Nanopore).
